# Unique miRNAs and their targets in tomato leaf responding to combined drought and heat stress

**DOI:** 10.1186/s12870-020-2313-x

**Published:** 2020-03-06

**Authors:** Rong Zhou, Xiaqing Yu, Carl-Otto Ottosen, Tingling Zhang, Zhen Wu, Tongmin Zhao

**Affiliations:** 1 Laboratory for Genetic Improvement of High Efficiency Horticultural Crops in Jiangsu Province, Institute of Vegetable Crop, Jiangsu Province Academy of Agricultural Sciences, Nanjing, Jiangsu China; 2 Department of Food Science, Aarhus University, Aarhus, Denmark; 3 Nanjing Agricultural University, Nanjing, Jiangsu China; 4 Shanghai Qingpu Vegetable Technology Promotion Station, Shanghai, China

**Keywords:** *Solanum lycopersicum* L., miRNAs, Degradome, Functional analysis, Combined abiotic stress

## Abstract

**Background:**

Both drought and heat stress are serious global problems, leading to agricultural production loss. MicroRNAs (miRNAs) play important roles in plant species responding to individual drought and heat stress. However, the miRNAs and mRNAs in association with combined drought and heat in crops like tomato remains unclear.

**Results:**

We studied the crosstalk of miRNAs and their target genes in tomato plants grown under simultaneous drought and heat stress that frequently happen in field conditions. In total, 335 known miRNAs representing 55 miRNA families and 430 potential novel miRNAs were identified in *Solanum lycopersicum* L. using small RNA deep sequencing. Through expression analysis, miRNAs in association with drought, heat and the combination of these were investigated. In total, 61, 74 and 37 miRNAs were differentially regulated for combination (of both stresses) vs control, combination vs drought and combination vs heat, respectively. Target genes with different expression levels were found using degradome sequencing, which were mainly involved in transcription factor activity, sequence-specific DNA binding, transcription, regulation of transcription, nucleus, DNA binding etc. The quantitative real-time polymerase chain reaction (qRT-PCR) results confirmed the accuracy of sequencing.

**Conclusions:**

Our study serves as valuable knowledge on how crop adapted to combined drought and heat stress by regulating miRNAs and mRNAs, which provide information for crop improvement to deal with future climate changes.

## Background

Drought stress due to deficit water supply was considered as the most destructive abiotic stress, which could happen at any crop growth stage and ultimately cause crop yield loss [1]. Heat stress caused by temperatures above optimum could induce complex response in plant [2]. More importantly, taking account into climate change and extreme weather events, the frequency of co-occurrence of drought and heat has increased especially during the summer period [3, 4].

MicroRNAs (miRNAs) in plant are a class of endogenous non-coding RNAs with about 21 nucleotides (nt) [5]. Due to extensive complementarity between miRNAs and target genes, the miRNA regulates gene expression by target cleavage in plants [5]. The miRNAs-mRNAs relationship has been widely identified by dengradome sequencing in various crops, such as tomato [6] and orchard grass [7]. Active response of miRNAs and mRNAs in plants has been widely associated to individual abiotic stress, including drought condition [7, 8] and high temperature [6]. It was found that 111 miRNAs were predominantly expressed in wheat after dehydration caused by polyethylene glycol (PEG) solution for 12 h [8], while 41 miRNAs were responsive to 18 days of individual drought condition in orchard grass and 5950 genes were targeted by 487 miRNAs [7]. Moreover, 96 and 150 miRNAs exhibited different expression levels in heat-tolerant wild tomato at moderately (33 °C) and acutely elevated temperature (40 °C) for 8 h [6]. Altogether 57 conserved and 41 novel miRNAs showed different expression levels in *Betula luminifera* treated by 0.5 h and 4 h of 45 °C [9]. However, the biological and molecular mechanisms of miRNAs in crop responding to combined stress are not well understood.

Tomato, as a model crop to investigate physiological and molecular response for scientific research, is sensitive to both drought and heat [10, 11], leading to a limitation on its growth and yield in semi-arid/arid region accompanied by heat waves. Compared with physiological and biochemical response, the research on genetic response in tomato to combined drought and heat is limited. For instance, simultaneous application of heat and drought caused both shared and unique response in tomato in the aspect of gas exchange, chlorophyll fluorescence, carbohydrate and reactive oxygen species (ROS) [10, 11]. Transcriptome analysis of *Arabidopsis* at combined drought and heat stress showed a unique pattern of defense response [12]. However, the regulatory mechanism between miRNAs and mRNAs in tomato subjected to combined stress and the crosstalk between individual and combined stress at post-transcriptional regulation level needs to be clarified.

Melatonin, *N*-acetyl-5-methoxytryptamin, is a well-known hormone that played roles in plant response to abiotic stress [13, 14]. Endogenous melatonin could be triggered by both moderate drought (withdrawing water) and cold (2 ± 0.5 °C) conditions for 48 h, which was involved in drought priming and cold stress response in barley [13]. Martinez et al. (2018) indicated that melatonin regulated genes and enzymes that were related to oxidative stress and ROS detoxification to improve tomato tolerance at combined salinity and heat [14]. In this study, significantly increased melatonin concentration was regarded as a responsive signal in tomato to the applied stresses, since the important roles of melatonin in plants at abiotic stress [13, 14]. We detected miRNA using high-throughput sequencing (HTS) and identified the target genes through degradome analysis in tomato after 36 h of stress treatments with three biological replicates when the melatonin concentration significantly increased. We characterized the roles of the target genes with significantly different expression levels. The expression level of miRNAs and their targets were further validated by quantitative real-time polymerase chain reaction (qRT-PCR) analysis. We aimed to arouse the attention of miRNAs-mRNAs regulation in tomato when drought and heat co-occur. We will give an overview of the interactions between differentially expressed miRNAs and the target genes with melatonin as a signal in tomato at individual and combined stress. This will contribute to uncover the post-transcriptional regulatory mechanisms underlying combined stress response in crop.

## Results

**Fig. 1:**
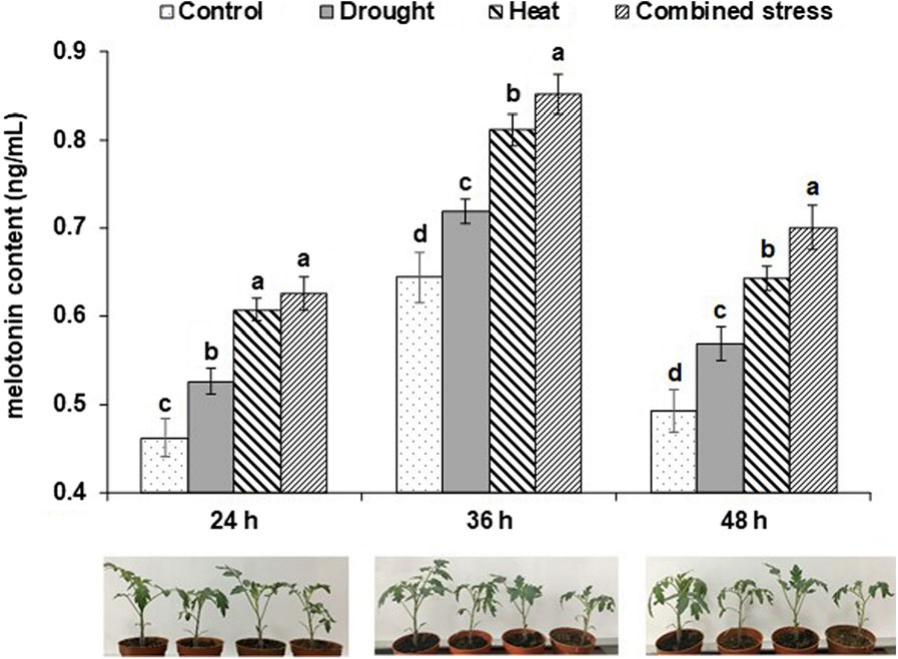
Melatonin content in tomatoes treated after control, drought, heat and combined stress for 24 h, 36 h and 48 h

As shown in Fig. 1, endogenous melatonin content in tomato at the three stresses were significantly higher than control at 24 h, 36 h and 48 h. At the three time points, heat stress induced significantly higher melatonin content as compared with drought stress (Fig. 1). At 36 h and 48 h, combined stress triggered significantly high accumulation of melatonin content in comparison with individual stress (Fig. 1). Thereby, we chose the samples taken at 36 h, which is the first time point when the four treatments exhibited significant difference between each other in melatonin content.

**Fig. 2:**
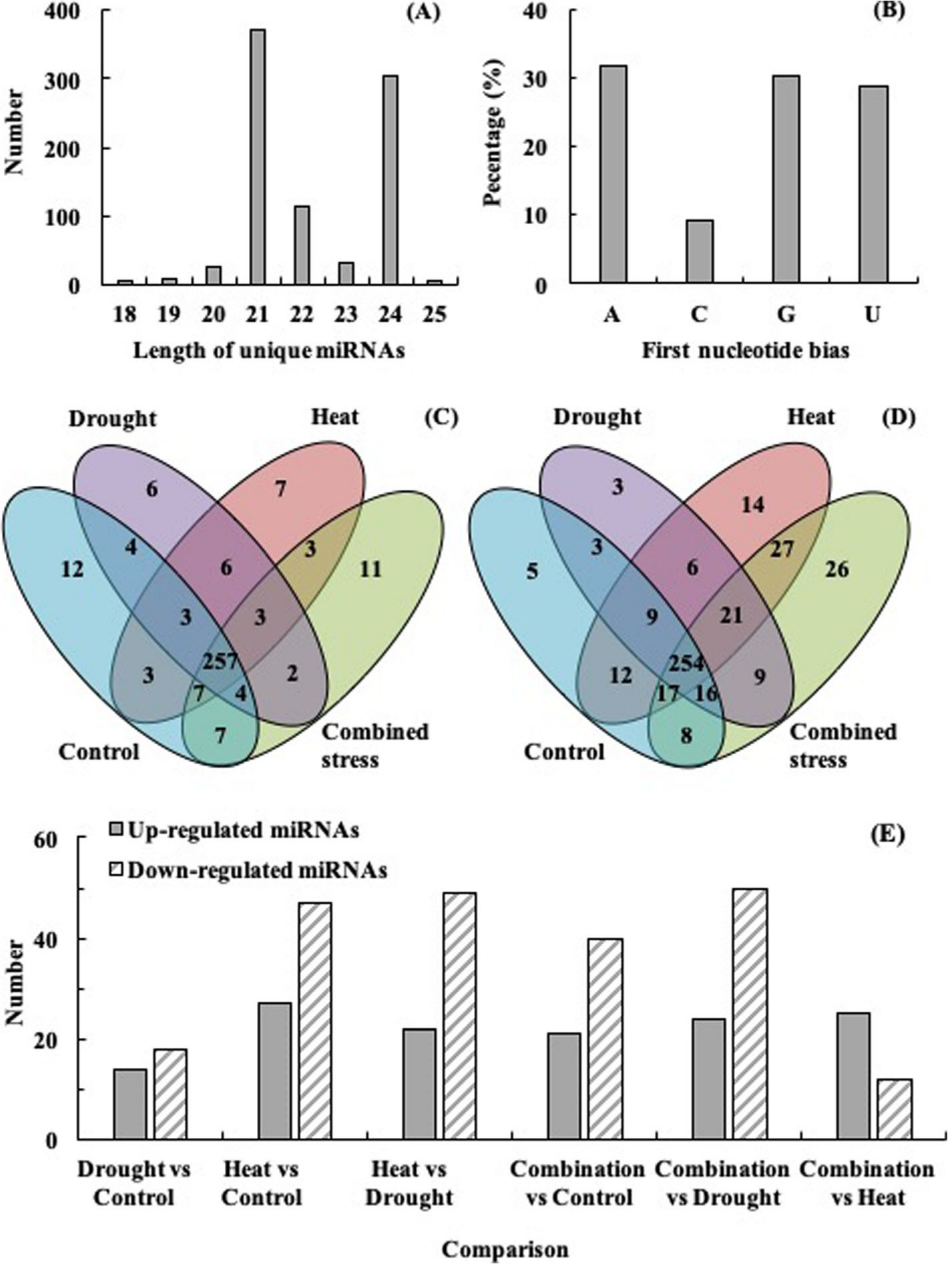
**a** Length distribution of the unique miRNAs; **b** percentage of first nucleotide bias in the unique miRNAs; **c** venn diagrams of conserved miRNAs; **d** venn diagrams of novel miRNAs and **e** significantly up−/down regulated miRNAs identified in tomatoes at control, drought, heat and their combination

There were 11,490,162, 10,175,417, 12,103,236 and 13,096,254 raw reads identified in tomatoes at control, drought, heat and their combination, respectively (Table 1). We filtered out 34.39, 32.01, 32.68 and 32.94% (reads/reads) redundant reads from the raw reads in tomatoes at the four treatments. Then, we obtained 7,539,067, 6,918,517, 8,148,031 and 8,781,914 valid reads in tomatoes at control, drought, heat and their combination, respectively (Table 1). In total, 765 miRNAs with 335 conserved and 430 novel miRNAs were genome-widely identified in tomatoes at the four treatments after removing the redundant miRNAs (Supplementary Table S1). The length of the identified miRNAs ranged from 18 nt to 25 nt with 21 nt (42.69%) as the most abundant category followed by 24 nt (34.98%) (Fig. 2a). Of the 765 miRNAs, 31.68, 30.19 and 28.78% started with adenine, guanine and uridine at the 5′-end, respectively (Fig. 2b). Among the miRNAs, 257 conserved and 254 novel miRNAs were shared in the four libraries (Fig. 2c, d). As compared with individual drought and heat, 28 (7 + 7 + 11 + 3) and 24 (7 + 4 + 2 + 11) conserved miRNAs were specifically identified in tomato at combined stress (Fig. 2c). For novel miRNAs, 78 (17 + 8 + 26 + 27) and 59 (8 + 16 + 9 + 26) miRNAs only existed in tomato at combined stress in comparison with individual drought and heat, respectively (Fig. 2d). There were 11 conserved and 26 novel miRNAs that were only identified in the tomato at combined stress among the four treatments (Fig. 2c, d).

**Table 1:** Data summary of small RNA sequencing in the tomatoes at control, drought, heat and combined stress

Library	Control		Drought		Heat		Combined stress	
	Number	Ratio (%)	Number	Ratio (%)	Number	Ratio (%)	Number	Ratio (%)
Raw reads	11,490,162	100.00	10,175,417	100.00	12,103,236	100.00	13,096,254	100.00
3ADT&length filter	1,594,860	13.88	1,390,187	13.66	1,791,717	14.80	1,617,878	12.35
Junk reads	67,008	0.58	61,220	0.60	65,770	0.54	80,198	0.61
Rfam	775,970	6.75	618,821	6.08	681,648	5.63	881,565	6.73
mRNA	1,730,428	15.06	1,363,539	13.40	1,613,061	13.33	1,991,808	15.21
Repeats	12,631	0.11	9719	0.10	12,502	0.10	17,989	0.14
valid reads	7,539,067	65.61	6,918,517	67.99	8,148,031	67.32	8,781,914	67.06
rRNA	636,934	5.54	506,964	4.98	519,914	4.30	674,582	5.15
tRNA	97,109	0.85	80,511	0.79	127,505	1.05	159,653	1.22
snoRNA	6747	0.06	5404	0.05	6252	0.05	8946	0.07
snRNA	2998	0.03	2265	0.02	1855	0.02	3307	0.03
other Rfam RNA	32,181	0.28	23,675	0.23	26,119	0.22	35,076	0.27

As compared with control, the expression levels of 32, 74 and 61 miRNAs were significantly changed in tomatoes at drought, heat and their combination (Fig. 2e, Supplementary Table S2). Moreover, as compared with single drought and heat, the expression levels of 74 and 37 miRNAs were significantly changed in tomatoes at combined stress (Fig. 2e, Supplementary Table S2). As compared with individual drought, the expression level of sly-miR172a-5p**_**stu and PC-298-5p significantly decreased and increased most (− 6.03 and 3.57 log_2_FC; FC means fold change) in tomato at combined stress, respectively (Supplementary Table S2). The expression level of PC-113-3p significantly decreased most (− 1.90 log_2_FC), while that of sly-miR160a-3p_stu significantly increased most (3.52 log_2_FC) in tomato at combined stress as compared with individual heat (Supplementary Table S2).

Based on degradome sequencing, 25,756,917, 25,537,341, 23,142,489 and 23,554,564 raw reads were identified in tomato at control, drought, heat and their combination, which corresponded to 4,346,794, 3,377,985, 3,056,688 and 2,869,388 unique raw reads, respectively (Table 2). The 22 target genes with significantly changed expression levels of 33 miRNAs for combination vs control were identified (Supplementary Table S3). Similarly, 20 target genes of 36 miRNAs and 14 target genes of 26 miRNAs showed significantly changed expression levels for combination vs drought and combination vs heat, respectively (Supplementary Table S3). One gene could be targeted by several miRNAs. For example, *RAP2–7* targeted by sly-miR172a-3p_csi, sly-miR172a_ath, sly-miR172e-3p_ath, sly-miR172k_gma, sly-miR172a and sly-miR172d-3p_stu in heat vs control and combination vs control (Supplementary Table S3). By contrast, several genes could be targeted by one miRNA, e.g. sly-miR396a-5p targeting *GRF3*, *GRF4* and *GRF8* (Supplementary Table S3). More importantly, one gene could be the target of single miRNAs. For instance, the *HSP22.7* was targeted by PC-287-5p, which was identified for heat vs control, heat vs drought, combination vs heat (Supplementary Table S3), indicating the specific regulation role of this miRNA and its target.

**Table 2:** Data summary of degradome sequencing in the tomatoes at control, drought, heat and combined stress

Library	Control		Drought		Heat	Combined stress		
	Number	Ratio (%)	Number	Ratio (%)	Number	Ratio (%)	Number	Ratio (%)
Raw reads	25,756,917	–	25,537,341	–	23,142,489	–	23,554,564	–
Unique raw reads	4,346,794	–	3,377,985	–	3,056,688	–	2,869,388	–
Transcript mapped reads	16,440,918	64.03	14,670,815	57.92	4,635,283	19.71	5,292,974	22.11
Unique transcript mapped reads	2,690,125	61.35	1,987,677	58.73	1,670,955	54.31	1,744,814	60.41
Number of input Transcript	35,768	–	35,768	–	35,768	–	35,768	–
Number of Covered Transcript	24,394	68.2	23,839	66.65	23,815	66.58	24,316	67.98

**Fig. 3:**
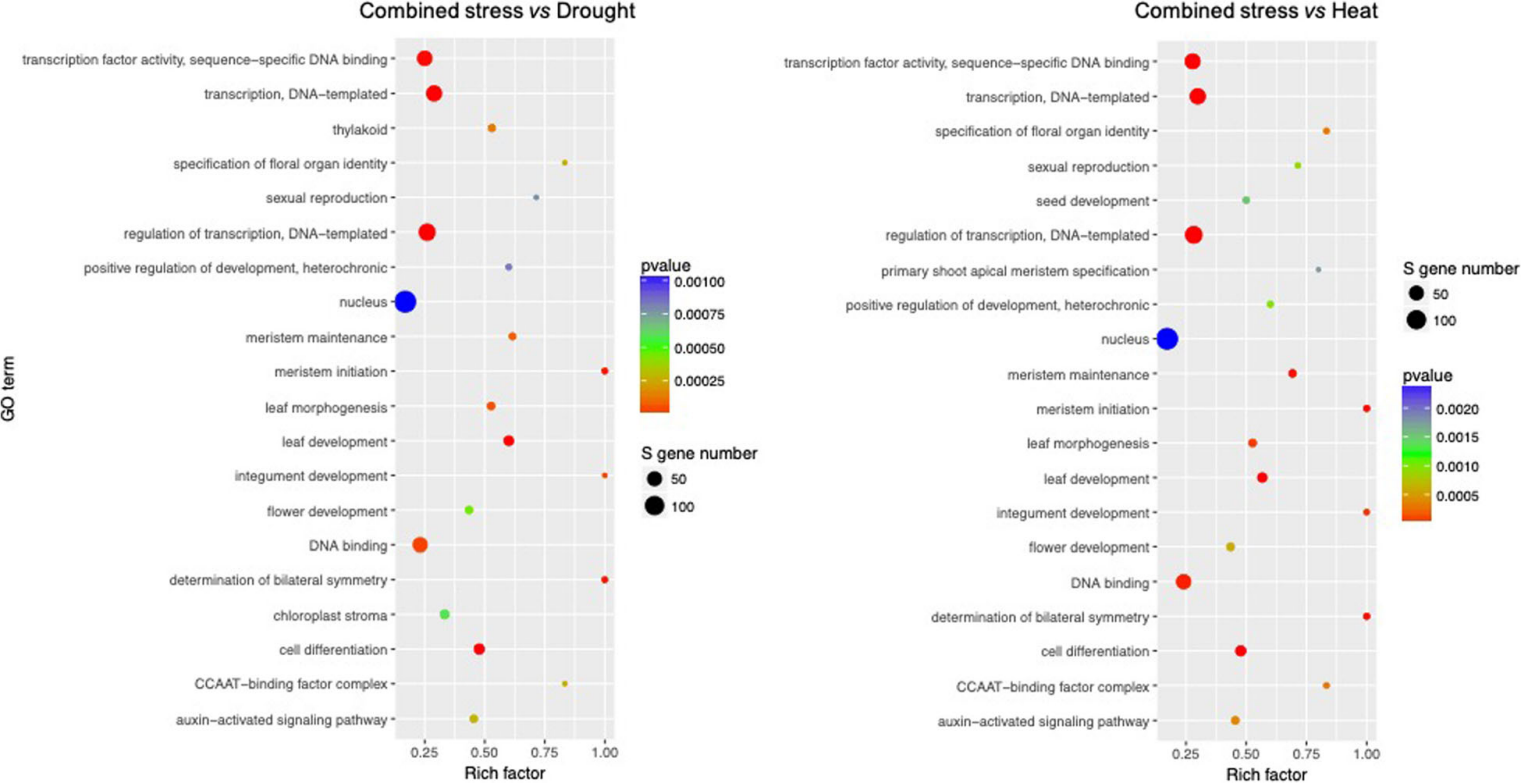
GO enrichment of target genes with significantly different expression level for combined stress vs drought and combined stress vs heat

To have an overview concerning the functions of the target genes with different expression levels, the GO enrichment analysis was conducted. As compared with control, individual stress exhibiting different expression levels primarily took effect in transcription factor activity, sequence-specific DNA binding, transcription, regulation of transcription, DNA binding etc. (Supplementary Figure S1). As compared with individual stress, the genes with different expression levels after combined stress mainly played roles in transcription factor activity, sequence-specific DNA binding, transcription, regulation of transcription, nucleus, DNA binding etc. (Fig. 3).

**Fig. 4:**
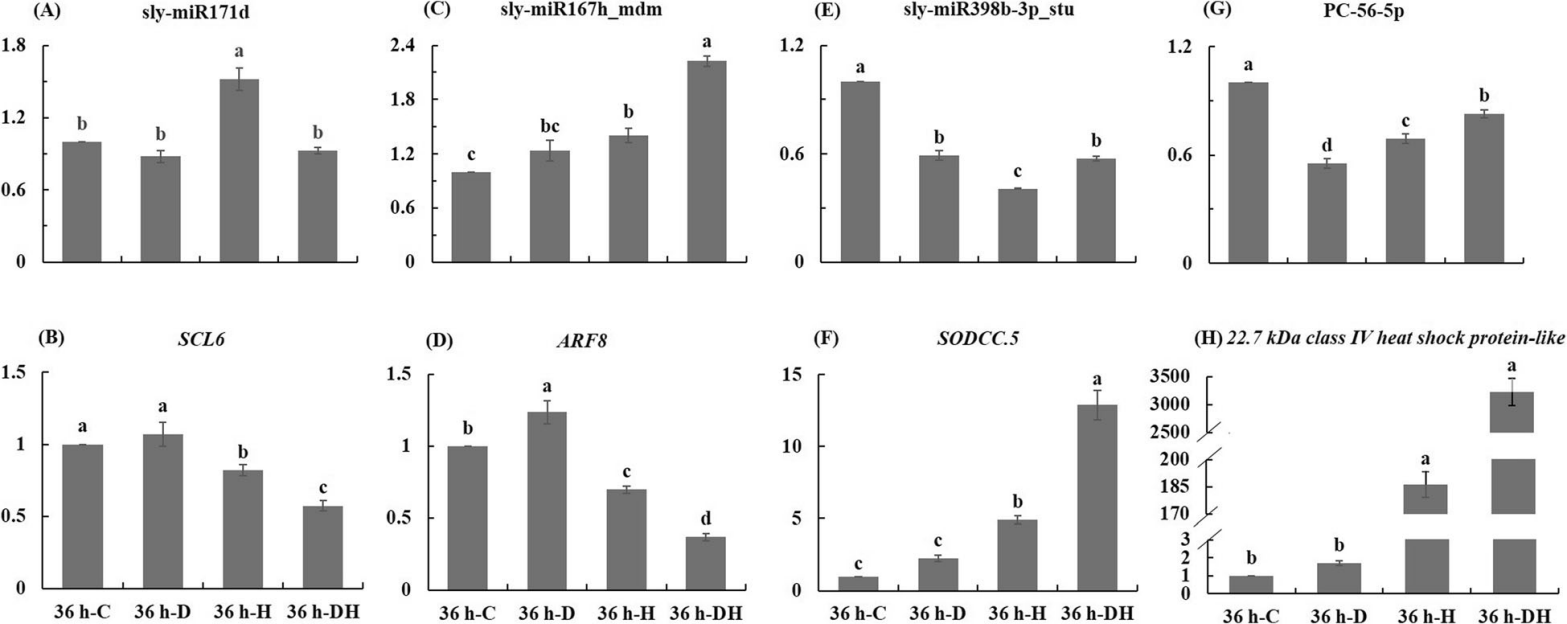
qRT-PCR validation of four miRNAs and four target genes

**Fig. 5:**
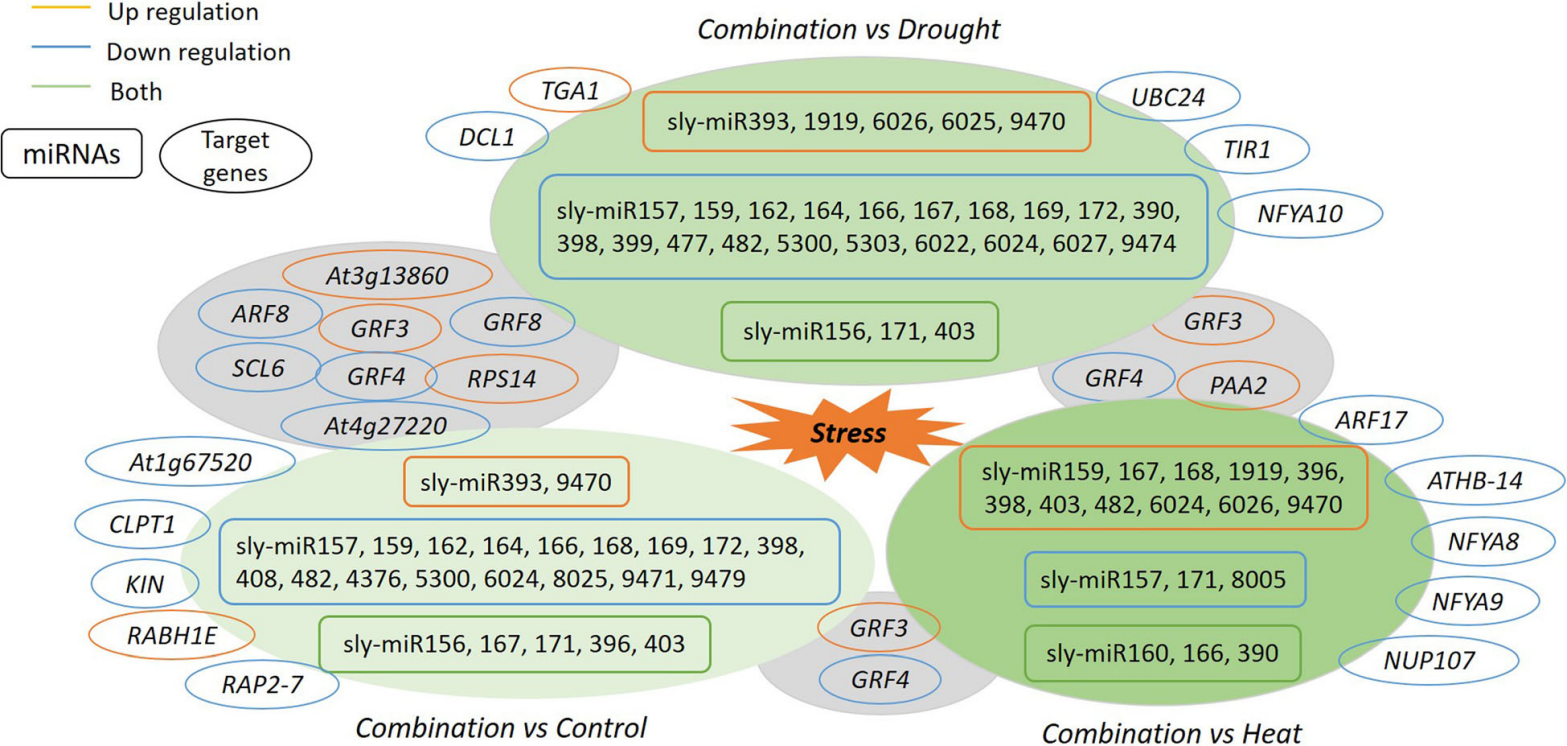
Network of miRNAs-mRNAs in tomato leaf at combined drought and heat stress as compared with control and individual stress. The miRNAs were shown in square box, while the target genes were shown in circle. The yellow color indicated that the miRNAs from the family /target genes were up-regulated; the blue color indicated that the miRNAs from the family /target genes were down-regulated; the green color indicated that the miRNAs from the family showed both up−/down- regulation. The shared response of the same genes was marked in the grey background

In order to verify the results of HTS, the expression levels of four miRNAs and four target genes were detected by qRT-PCR. The expression level of sly-miR171d significantly increased after heat treatment, while its target gene (*SCL6*) significantly decreased after heat and combined stress (Fig. 4a, b). The expression level of sly-miR167h_mdm was significantly higher after heat and combined stress than control, while its target gene (*ARF8*) was higher after drought but lower after heat and combined stress (Fig. 4c, d). The expression level of sly-miR398b-3p_stu and PC-56-5p significantly decreased after three stress conditions (Fig. 4e, g). The expression level of *SODCC.5*, the target gene of sly-miR398b-3p_stu, as well as the *22.7 kDa class IV heat shock protein-like*, significantly increased after heat and combined stress as compared with control (Fig. 4f, h). The comparison of miRNAs-mRNAs network in tomato leaf between individual and combined stress was conducted. The response of certain miRNAs (miR157, 159, 166, 167, 168, 398, 482 and 6024) in tomato at combined stress is unique, the expression level of which were different from all the other three treatments (Fig. 5). Moreover, combined stress up-regulated the target genes *GRF3* but down-regulated *GRF4* as compared with the other three treatments (Fig. 5).

## Discussion

The frequency and intensity of climate extreme events are increasing, e.g. naturally coupled drought and heat stress [4]. Understanding the underlying mechanisms of abiotic stress response is important for the breeding of tolerant crop cultivars [15]. Tomato developed dynamic and various responses at the physiological, metabolic and biochemical levels when drought and heat co-happened [10, 11]. Recent studies has widely reported the expression patterns of miRNAs in tomato responding to abiotic stress like high temperature [6] and biotic stress such as *Phytophthora infestans* [16] and *Botrytis cinerea* [17].

However, to date, expression profiles of miRNAs and their targets in tomato responding to combined drought and heat stress remain unexplored. Thereby, miRNAs-mRNAs regulatory mechanisms need to be thoroughly investigated in tomato at individual and combined stress to understand their interaction.

### Expression pattern of miRNAs in tomato leaf responding to individual and combined stress

In this study, a combination of miRNA sequencing and degradome analysis was used to characterize the miRNAs-mRNAs expression profiles. Previous evidence showed that abiotic stresses, such as drought and temperature extremes, could promote endogenous melatonin accumulation in plant [13, 18]. Thereby, with significant increases in endogenous melatonin content as a signal, the leaves from tomato plant after 36 h of treatments were used for HTS and degradome analysis.

Of the miRNAs, 21-nt miRNAs (42.7%) were most abundant followed by 24-nt miRNAs (35.0%) (Fig. 2a), representing the predominant length of mature miRNAs in plant species, which was also reported by Zhou et al., (2016) [6] and Ji et al. (2018) [7]. Usually, the mature miRNA were incorporated into argonaute (AGO) to form the RNA-induced silencing complex (RISC), resulting in mRNAs cleavage in plant [5, 19]. AGO1 preferentially recruit miRNAs with 5′ terminal uridine, whereas AGO2 and AGO4 harbors miRNAs that favor the 5′ terminal adenosine [20]. The 5′ terminal nt of miRNA is a crucial characteristic that affects miRNAs biological activity and function [20]. Therefore, we detected the 5′ terminal nt distribution of miRNAs. The miRNAs identified in this study could play crucial roles by cleaving mRNAs indicated by 31.68 and 28.78% miRNAs with adenosine and uridine as 5′ terminal nt, respectively (Fig. 2b). A total of 335 known miRNAs distributed across 55 families and 430 potential novel miRNAs were identified. In addition, 32, 74 and 61 miRNAs were differentially expressed for drought vs control, heat vs control and combination vs control respectively.

On one hand, miRNAs in association with individual drought and heat have been reported in various plants including tomato [6, 21]. In accordance with the results from Candar-Cakir et al. (2016) [21], drought condition did affect miR166 family with both up- and down-regulated miRNAs but not affect miR165 family in upground tissue from tomato seedling. Candar-Cakir et al. (2016) found that the expression of miRNAs from miR398 family were generally lower at drought treatment than control in upground tissues [21]. This is not the same case as our study, which could be explained by difference in the duration, intensity and approach of the drought stress as well as cultivar specificity [22]. Similarly, miR156 and miR167 family was induced by exposure to both moderately and acutely elevated temperatures (33 and 40 °C) [6]. However, sly-miR167b-3p_stu, sly-miR167d-3p_stu and sly-miR167c-3p_stu were concurrently down-regulated (− 1.65, − 0.76 and − 3.74 log_2_FC) at high temperature condition here. Meanwhile, sly-miR156a_stu and sly-miR156g-p3_nta showed opposite trends (0.92 and − 3.71 log_2_FC) at heat stress. Therefore, the members from the same miRNA family may differently respond to abiotic stress including drought and heat, despite the similarity of each member [21, 23]. MiR159 is a well-known responsive miRNA to heat stress [22]. We found that sly-miR159a-5p_gma and sly-miR159-p5 were down-regulated (− 4.16 and − 3.69 log_2_FC) in tomato at high temperature, which was in accordance with the results in wheat cultivar ‘Ofanto’ [22]. On the other hand, we found that combined stress provoked specific miRNAs response with 74 and 37 showing up/down regulation in comparison with drought and heat. Together with our previous results [10, 11], the combined of drought and heat induced unique response in the aspect of physiology, ROS and miRNAs in tomatoes.

### Crosstalk of miRNAs-mRNAs in tomato at individual and combined stress

Previous studies unveiled the mechanisms between tomato response to individual heat or drought stress and miRNAs-mRNA [6, 24]. For instance, 138 conserved miRNAs were targeted by 163 genes and eight novel miRNAs were targeted by 10 genes in tomato heat response based on dengradome sequencing [6]. There were 1936 putative target genes for the differentially-expressed miRNAs in tomato drought response [24]. However, the roles of miRNAs underlying the complex combined stress response in crop remain unexplored. In degradome analysis, a total of 22 target genes with changed expression levels were identified for 33 miRNAs in the case of combination vs control. Moreover, 20 target genes of 36 miRNAs and 14 target genes of 26 miRNAs were up/down regulated for combination vs drought and combination vs heat, respectively.

Plant hormone is an important component in signal pathways, which plays a role in increasing plant tolerance to abiotic stress [25, 26]. *SPL2* targeted by miR156 family (sly-miR156a, sly-miR156a_stu, sly-miR156f-p5_stu and sly-miR156e_mtr) were detected in drought vs control, which was involved in plant hormone signal transduction (Supplementary Table S3). In addition, miR398 was down-regulated on different levels in tomato treated by *P. infestans* infection, salt and drought conditions [27]. *SODCC.5* played a role in copper/zinc superoxide dismutase that can protect plant from superoxide radicals as a scavenger enzyme of ROS [28]. We found that *SODCC.5* targeted by sly-miR398b-3p_stu (− 1.20 log_2_FC) was up-regulated (2.80 log_2_FC) for heat vs drought (Supplementary Table S3), indicating the differently regulatory mechanisms of copper/zinc superoxide dismutase in tomato induced by heat and drought conditions. This corresponded to our previous study that higher SOD activity in two tomato cultivars at heat stress than drought stress on day 6 [11].

Stomata and pore length, net photosynthesis and starch content significantly decreased caused by drought and combined stress in three tomato cultivars [10]. A decreased expression level of *Solyc02g086820.3.1* (− 5.65 and − 5.23 log_2_FC) targeting by PC-326-3p was found in heat vs drought and combination vs drought, the function of which was mainly involved in carbonate dehydratase activity, chloroplast, response to carbon dioxide, regulation of stomatal movement and stomatal complex development, carbon utilization, photosynthesis (Supplementary Table S3). This suggested that the alteration of stomatal, gas exchange and carbohydrate metabolism could be partly the result of differential expression of the targets that were regulated by certain miRNAs in tomato.

Auxin response factor (*ARF*) played important roles in stress adaption and physiological mechanisms mediated by auxins [29]. We found that *ARF8* targeted by sly-miR167h_mdm and sly-miR167a-5p_ath showed decreased expression levels for heat vs control (− 2.16 log_2_FC), heat vs drought (− 2.35 log_2_FC), combination vs control (− 2.37 log_2_FC), combination vs drought (− 2.56 log_2_FC) (Supplementary Table S3). Similarly, the expression level of *ARF17* targeted by sly-miR160a-5p_stu, sly-miR160a_vvi and sly-miR160a decreased for combination vs heat (− 1.39 log_2_FC) (Supplementary Table S3). Therefore, the miR160 and miR167 restrained ARF-mediated expression of auxin responsive genes, leading the attenuated growth and development in plant at abiotic stress [30], especially for combined stress here. Previous study documented that miR393 downregulated transport inhibitor response1 (*TIR1*) in plant at abiotic stress [30]. Accordingly, *TIR1* (− 2.98 log_2_FC) targeted by sly-miR393_ghr (0.97 log_2_FC) showed opposite trend in expression level for combination vs drought (Supplementary Table S3). This suggested that the regulation of *TIR1* by miR393 might play key roles in tomato at combined stress as compared with drought. Growth-regulating factors (*GRFs*), plant-specific transcription factors, can coordinate plant growth processes at abiotic stress and has been identified for their roles in leaf development [31]. *GRFs* played unique roles in the response of plant to combined drought and heat, since *GRF3* and *GRF4* were significantly regulated through miR396 in tomato at combined stress in comparison with all the other three treatments (Fig. 5).

The combination of heat and drought imposes a specific set of physiological restrains and unique transcriptome responses on various plant species, which were not altered by applying individual stresses [10, 32–34]. We found that miRNAs-mRNAs work together and provoke combinatorial effectors when abiotic stresses co-happened. Competing endogenous RNAs (ceRNAs) with shared miRNAs recognition elements (MREs), such as mRNAs, lncRNAs and circRNAs, compete for miRNA binding and regulate each other [35]. Dynamic balance of miRNAs, mRNAs and other ceRNAs is crucial for regulating cellular homeostasis in plant under stress conditions. The regulatory network between miRNAs-mRNAs was elucidated in tomato responding to combined stress in this study. It is important to take into consideration that the ceRNAs including lncRNAs and circRNAs to gain knowledge on crop response to dynamic climates in our future work.

## Conclusions

Through HTS, qRT-PCR and bioinformatics analyses, we performed, for the first time, one integrative analysis of the potential miRNA-mRNA regulatory networks in tomato responding to individual and combined stress. We found that 11 conserved and 26 novel miRNAs specifically existed in tomato treated by combined drought and heat as compared with control and individual stress. The miRNAs-mRNAs formed complex regulation networks in tomato’s response to combined stress. The target genes primarily participated in transcription factor activity, sequence-specific DNA binding, transcription, regulation of transcription, nucleus, DNA binding other pathways. The qRT-PCR verification corresponded to the sequencing results. In conclusion, our findings provide new knowledge on miRNA regulation of target expression in tomato at combined stress, which serve as a useful resource for further analysis of the interactions between miRNAs and target genes in crops responding to co-concurrent abiotic stresses due to climate change.

## Methods

### Plant culture and sample collection

Seeds of the popular tomato cultivar ‘Jinlingmeiyu’ from Jiangsu Province Academy of Agricultural Sciences were sown in plug trays. This cultivar was not tolerant to individual heat, drought and their combination, especially severe drought and combined stress. The plants were cultivated in climate chambers (RDN-560E-4, Dongnan Instrument Co, Ltd., Ningbo, China) with 50–60% relative humidity. The air temperature was 26/18 °C (14 h daytime from 6:00–20:00). Light intensity was 300 μmol m^− 2^ s^− 1^ photosynthetic photon flux density (PPFD) (white LED light source, Dongnan Instrument Co, Ltd., Ningbo, China). The plants were irrigated by water every 5 days. After 15 days, the plants were irrigated by nutrient solution based on the Japanese Garden test formula every 3 days. The drought treatment started by withholding water for half of the 24-day-old plants. After 3 days, the plants with/without irrigation were treated at 26 °C as control and drought treatment; the plants with/without irrigation were treated at 38 °C as heat and combined stress treatment. The treatments lasted for 48 h during the light condition. The first fully expanded leaf from top was taken at 24 h, 36 h and 48 h after treatments and kept at − 80 °C in Jiangsu Province Academy of Agricultural Sciences, Jiangsu, Nanjing.

### Determination of endogenous melatonin content

The leaf samples taken at 24 h, 36 h and 48 h after the four treatments were used to determine the endogenous melatonin content with three replications per treatment. The leaf tissues were homogenized and the melatonin was extracted using plant MT ELISA kit (Lanpai Bio, Shanghai, China). The 10 μL testing sample and 40 μL sample diluent were added in the wells with 50 μL standard as control. Afterwards, 100 μL horseradish peroxidase were added to each well. The samples were covered with adhesive strip followed by incubation for 1 h at 37 °C. The samples were aspirated and washed by filling the wells with 400 μL wash solution using a squirt bottle for five times. The 50 μL chromogen solution A and 50 μL chromogen solution B were mixed with the samples, followed by incubation for 15 min at 37 °C in darkness. The 50 μL stop buffer was added and the plate were gently tapped to ensure thorough mixture. The reads were taken record at 450 nm within 15 min using a microtiter plate reader (F50 TECAN SUNRISE, Germany).

### Genome-wide miRNAs and their targets identification

Based on the results of endogenous melatonin contents in tomato at the four treatments at different time point, the samples taken at 36 h were used for HTS with three biological replicates by LC-BIO (Hangzhou, China).

The samples were used to extract sRNAs using TruSeq Small RNA Sample Preparation Kits (Illumina, San Diego, USA). The cDNAs were obtained through reverse transcription of sRNAs library after isolation and ligation. Following the purification, the cDNAs were sequenced using Illumina Hiseq2500 (LC-BIO, Hangzhou, China). Afterwards, redundant sequences were removed to obtain valid sequences by blasting against Rfam (http://rfam.janelia.org), repeat database (http://www.girinst.org/repbase) and Solanaceae Genome Network database (http://sgn.cornell.edu/). The valid sequences were aligned with the known mature pre-miRNAs and miRNAs in plants according to miRbase 21.0. The sequences that mapped to mature miRNAs in hairpin arms and other arm of precursor hairpin were considered as conserved and novel miRNAs.

Total RNAs were extracted for degradome analysis using Trizon reagent (Ivitrogen, CA, USA). The samples were blended with biotinylated random primers, captured by beads and ligated to 5′ adaptors. The ligated sequences were then reversely transcribed to obtain the first-strand cDNAs. After purification, digestion, ligation and repurification, the degradome library was sequenced using the Illumina Hiseq2500 (LC-BIO, Hangzhou, China). The data from degradome sequencing were analysed using CleaveLand 3.0. The degradome density file was generated by basting degradome sequences with.

ftp://ftp.solgenomics.net/tomato_genome/annotation/ITAG3.2_release. Targetfinder was applied for the prediction of the target genes. Following the associative calculation of the target genes based on degradome density file and prediction, the common target genes were found and regarded as the final target genes.

### Functional analysis

Gene ontology (GO) annotation was performed according to http:/geneontology.org/. The GO enrichment analysis of the target genes with different expression levels between the libraries was conducted using the formula as follows:

$$ P=1-{\sum}_{i=0}^{S-1}\frac{\left({}_i^B\right)\left({}_{TS-i}^{TB-B}\right)}{\left({}_{TS}^{TB}\right)} $$

B: number of mRNAs with functions; S: number of mRNAs with functions corresponding to miRNAs; TB: mRNAs total number; TS: number of mRNAs corresponding to miRNAs.

### qRT-PCR verification

The expression levels of four miRNAs (sly-miR171d, sly-miR167h_mdm, sly-miR398b-3p_stu and PC-56-5p) and four target genes (*SCL6*, *ARF8*, *SODCC.5* and *22.7 kDa class IV heat shock protein-like*) were verified using qRT-PCR. The RNAs was extracted using Trizol reagent (Invitrogen, CA, USA). The RNAs were reversely transcribed using PrimeScript 1st strand cDNA Synthesis Kit (TaKaRa, Dalian, China). The transcription condition for miRNAs was 25 °C for 10 min, 42 °C for 30 min, 85 °C for 5 min, while that for target genes was 30 °C for 10 min, 42 °C for 30 min, 95 °C for 5 min. The SYBR Premix Ex Taq were provided by TaKaRa, Dalian, China. The qRT-PCR reaction condition was 95 °C for 30 s once, 95 °C for 5 s and 60 °C for 20 s by 40 cycles, 72 °C. The primers for miRNAs and mRNAs were shown in Supplementary Table S4 and Supplementary Table S5, respectively. U6snRNA and Actin was the reference for miRNAs and mRNAs, respectively, according to our previous report [6]. The expression levels were calculated by the 2^−ΔΔCt^ method with three biological and three technical repetitions.

### Data analysis and access

Analysis of variance (ANOVA) for the melatonin content, four miRNAs and four target genes expression levels in tomatoes at the four treatments were performed using SPSS 16.0 (SPSS Inc., Chicago, IL, USA). The expression levels of the miRNAs and their target genes were compared using a chi-square test with *P* ≤ 0.05 as threshold value.

## Supplementary information

**Additional file 1: Figure S1.** GO enrichment of target genes with significantly different expression level for drought vs control, heat vs control and combined stress vs control.

**Additional file 2: Table S1.** Summary of the miRNAs identified in tomatoes at control, drought, heat and combined stress

**Additional file 3: Table S2.** Summary of the differentially expressed miRNAs identified in tomatoes at control, drought, heat and combined stress

**Additional file 4: Table S3.** Target transcripts with different expression levels cleaved by miRNAs in the tomatoes at control, drought, heat and combined stress by degradome analysis

**Additional file 5: Table S4.** Specific qRT-PCR primers for the miRNAs

**Additional file 6: Table S5.** Specific qRT-PCR primers for the target genes

## Data Availability

All data of our study was submitted to GEO (Gene Expression Omnibus) with the accession number of GSE121089 (https://www.ncbi.nlm.nih.gov/geo/query/acc.cgi?acc=GSE121089).

## Abbreviations

AGO: Argonaute
ANOVA: Analysis of variance
ceRNA: Competing endogenous RNA
GEO: Gene expression omnibus
GO: Gene ontology
HTS: High-throughput sequencing
miRNAs: MicroRNAs
MRE: MiRNAs recognition element
nt: nucleotide
PPFD: Photosynthetic photon flux density
qRT-PCR: Quantitative real-time polymerase chain reaction
RISC: RNA-induced silencing complex
