# Supplementary figures and images for: Unique miRNAs and their targets in tomato leaf responding to combined drought and heat stress
Source: BMC Plant Biol. 2020 Mar 6;20:107. doi: 10.1186/s12870-020-2313-x (PMC7060562; doi:10.1186/s12870-020-2313-x)

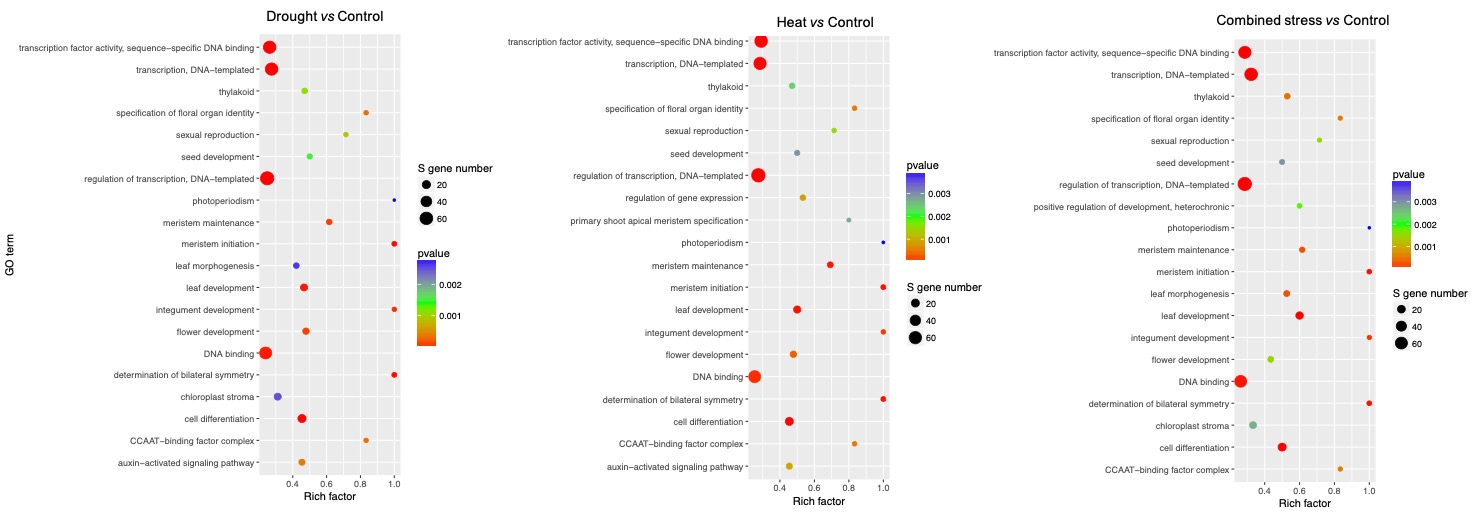

Supplement: Supplementary file 1 — Additional file 1: Figure S1. GO enrichment of target genes with significantly different expression level for drought vs control, heat vs control and combined stress vs control. [file 12870_2020_2313_MOESM1_ESM.jpg]
